# Occurrence and outcomes of possible superadded infections in older adults with COVID-19—cohort study

**DOI:** 10.1007/s41999-022-00675-9

**Published:** 2022-07-13

**Authors:** Jimmy Bilan, Ken Aggrey, Terence J. Quinn, Jane Lumsden, Kirsty Colquhoun

**Affiliations:** 1 School of Medicine, Dentistry and Nursing, University of Glasgow, Glasgow, UK; 2 Institute of Cardiovascular and Medical Sciences, University of Glasgow, Glasgow, UK; 3 Department of Medicine for the Elderly, Glasgow Royal Infirmary, Glasgow, UK

**Keywords:** COVID-19, Geriatric medicine, Superadded infections, Older adults

## Abstract

**Purpose:**

Current guidance discourages use of antibiotics in COVID-19. However, in older adults, superadded infection may be common and require treatment. Our aim was to investigate the occurrence and outcomes from possible superadded infections, occurring within 2 weeks of hospitalization, in older adults with COVID-19.

**Methods:**

This was a single centre, observational cohort study. We collected data from patients admitted to older adult wards who had tested positive for the Sars-CoV-2 virus on viral PCR between 1st October and 1st December 2020. The primary outcome was inpatient death occurring within 90 days of COVID-19 diagnosis. The secondary outcome was length of stay in hospital. Associations were described using univariable and multivariable models, and time to event data.

**Results:**

Of 266 patients with COVID-19, 43% (115) had evidence of superadded infections (91 with positive bacterial cultures and 36 instances of radiological lobar consolidation). Patients with superadded infections were more likely to die (45.2 versus 30.7%, *p* = 0.020) and had an increased length of stay (23 versus 18 days, *p* = 0.026).

**Conclusions:**

Recommendations to avoid antibiotics in COVID-19 may not be applicable to an older adult population. Assessing for possible superadded infections is warranted in this group.

**Supplementary Information:**

The online version contains supplementary material available at 10.1007/s41999-022-00675-9.

## Key summary points

**Aim:**

To investigate the outcomes and prevalence of superadded infections in older adults with COVID-19.

**Findings:**

Possible superadded infection is common in older adults hospitalized with COVID-19 and was associated with longer inpatient stay and higher mortality.

**Message:**

Clinicians should have a low threshold to assess for other sources of infection in older adults with COVID-19.

**Supplementary Information:**

The online version contains supplementary material available at 10.1007/s41999-022-00675-9.

## Introduction

Coronavirus Disease 2019 (COVID-19) remains a major threat to global health [1]. The systemic inflammatory response to Sars-CoV-2 can be substantial and clinical presentations of COVID-19 are varied. Thus, discriminating between symptoms of COVID-19 and symptoms of co-infection is a particular clinical challenge. This is especially true in the older adult, where non-specific presentations of disease are common to both COVID-19 and other infections [2].

Estimates of superadded infection (SAI) in COVID-19 have generally focused on bacterial pneumonia in populations of predominantly middle-aged patients. In this context, SAI is relatively infrequent, with reports that 3.5% of patients have co-infection on presentation and 14.3% develop a secondary infection during their admission [3]. The burden of all-cause SAI in older adults has not received similar attention.

With a relatively low occurrence of SAI, yet high rates of antibiotic prescribing in COVID-19, recommendations have focused on limiting the use of antibiotics [4]. This guidance is based on research from predominantly young and middle-aged cohorts. There remains equipoise as to whether this message is also relevant in older adults, especially older adults with frailty. This group are more likely to have multiple acute issues that precipitate a hospital admission and are particularly prone to infection [5]. SAI may be an important contributor to the high mortality observed in older COVID-19 patients, however, inappropriate antibiotic use risks antibiotic-related adverse events and antimicrobial resistance [6].

Our aim was to investigate the occurrence of possible SAI occurring early in hospitalization with COVID-19 of older adults. As secondary aims we assessed for differences at baseline and in outcomes between those with and without possible SAI and described patterns of microbiology investigations.

## Methods

### Design

This was an observational cohort study. The inception point was diagnosis of COVID-19. Data were recorded directly, using clinical case records as necessary, and cross-checked with electronic data systems. Where data were missing or unclear, clarification was sought from the treating clinical team.

As the study involved anonymous recording of data collected in the clinical routine, ethical review was considered unnecessary, authority to conduct the study was granted by the Health Research Authority (20/HRA/1898). Our approvals allowed us to work with anonymized routine clinical data, with no requirement for individual consent. Reporting followed the Strengthening the Reporting of Observational studies in Epidemiology (STROBE) guidelines [7].

### Setting

The study was based in a single centre University Hospital—the Department of Medicine for the Elderly, Glasgow Royal Infirmary, UK. The hospital admits older adults from an urban area with high levels of socioeconomic deprivation. The Department offers acute care but patients requiring level two care (i.e., settings offering additional staffing and usually single organ support or monitoring, often termed a high dependency unit) or higher would not be directly admitted. Data from 1st October to 1st December 2020 were used, representing the second major wave of COVID-19 infection in Glasgow.

### Participants

Participants were sequential hospital inpatients in the older adult service, who had tested positive for the Sars-CoV-2 virus on viral PCR during admission.

### Exposure

The exposure of interest was possible SAI within 14 days of Sars-CoV-2 diagnosis. SAI was defined as a positive microbiology culture of any site or evidence of single lobar consolidation on chest radiography. This definition was felt to represent conditions where treatment for co-infection could be reasonably considered. SAI status was recorded at the patient level. If a patient had, for example, two different positive specimens, this was treated as two instances of SAI in one patient. The frequency of different strains of bacteria measured by specimen culture was recorded and classified depending on culture type using categories of urine, blood, sputum, or other cultures (for example stool cultures).

### Variables

Other variables measured include age (years), sex, CRP, albumin, use of dexamethasone, and frailty. Frailty in our patient population was recorded using the 9-point clinical frailty scale [8]. Prior medical history such as diabetes, coronary artery disease, hypertension, chronic obstructive pulmonary disease, congestive cardiac failure, and pneumonia were recorded and defined by the treating clinical teams.

### Outcomes

The primary outcome was inpatient death within 90 days of COVID-19 diagnosis (*90-day mortality*). Secondary outcome was length of stay in hospital (from COVID-19 diagnosis until discharge inclusive). For those patients who died in hospital, the length of stay until death was recorded.

### Analyses

Characteristics of study participants were described with summary statistics appropriate to the data. Data regarding nominal characteristics of the study participants were described using frequencies and percentages. All normally distributed data were described using mean and standard deviation (SD). Non-normally distributed quantitative variables used median and interquartile ranges (IQR). Normality of data was verified using the Shapiro–Wilk test. Comparisons were performed using the Mann–Whitney *U* test or Chi-Square test as appropriate.

To identify characteristics associated with mortality, an initial univariable analysis was performed on available demographic (age, gender), laboratory (CRP, albumin) and pharmacological data (dexamethasone use) for those with and without possible SAI. Choice of variables for inclusion in subsequent multivariate analyses (by logistic regression and by a supplementary Cox regression) was based on biological plausibility and previous literature. Variables showing significance of *p* ≤ 0.25 in univariate analysis and those deemed clinically important qualified for inclusion in the multivariate model [9]. We assessed mortality occurring within 90 days and as a post hoc exploratory analysis, also assessed mortality within 30 days. Survival data, censored at 90 days, were analyzed using Kaplan Meier methods with Mantel Cox Log Rank testing. To account for the potential bias on length of stay for early mortality, we re-ran our length of stay analyses excluding inpatient mortality. Statistical significance (two-tailed) was defined as *p* ≤ 0.05. All analyses were performed using SPSS (version 28, IBM Corp, USA).

## Results

### Participants

The median age was 81 years in both SAI and NSAI groups, with the SAI group being 59% female and the NSAI group being 44% female. All participants had been admitted to the Department of Medicine for the Elderly during the recruitment window. Of 266 inpatients with PCR evidence of COVID-19 confirmed within the recruitment window, 43% (115) had evidence of possible SAI based on our criteria (91 bacterial cultures and 36 instances of radiological lobar consolidation). The group with possible SAI had marginally higher CRP levels and use of dexamethasone, but neither difference was significant (Table 1).

**Table 1:** Patient demographics, clinical features, and pre-existing diseases

	All patients	Possible SAI	No SAI detected	*p*
Total	266 (100%)	115 (43%)	151 (57%)	
Age (years)	81 (11)	81 (11)	81 (12)	0.528
Females	134 (50%)	68 (59%)	66 (44%)	0.013
CRP (μg/mL)	36 (75)	39 (80)	34 (72)	0.354
Albumin (g/L)	32 (6)	32 (6)	32 (7)	0.680
Dexamethasone	143 (54%)	68 (59%)	75 (50%)	0.125
Time from admission to diagnosis of COVID-19	239 (90%)	17.5 (128)	6 (19)	0.006
Diabetes	88 (33%)	40 (35%)	48 (32%)	0.607
Coronary artery disease	87 (33%)	34 (30%)	53 (35%)	0.341
Hypertension	158 (59%)	71 (62%)	87 (58%)	0.498
Chronic obstructive pulmonary disease	58 (22%)	23 (20%)	35 (23%)	0.534
Congestive cardiac failure	44 (17%)	18 (16%)	26 (17%)	0.733
Pneumonia (previous)	29 (11%)	11 (10%)	18 (12%)	0.541

*IQR* Interquartile range

Data are *n* (%) or median (IQR)

There was no significant difference in the frequency of comorbidities recorded at presentation between both patient groups (Table 1). Patients within our cohort with possible superadded infection had an average clinical frailty scale score of 5.41 compared to 4.93 in those without evidence of superadded infection (*p* = 0.005).

### Outcomes

**Fig. 1:**
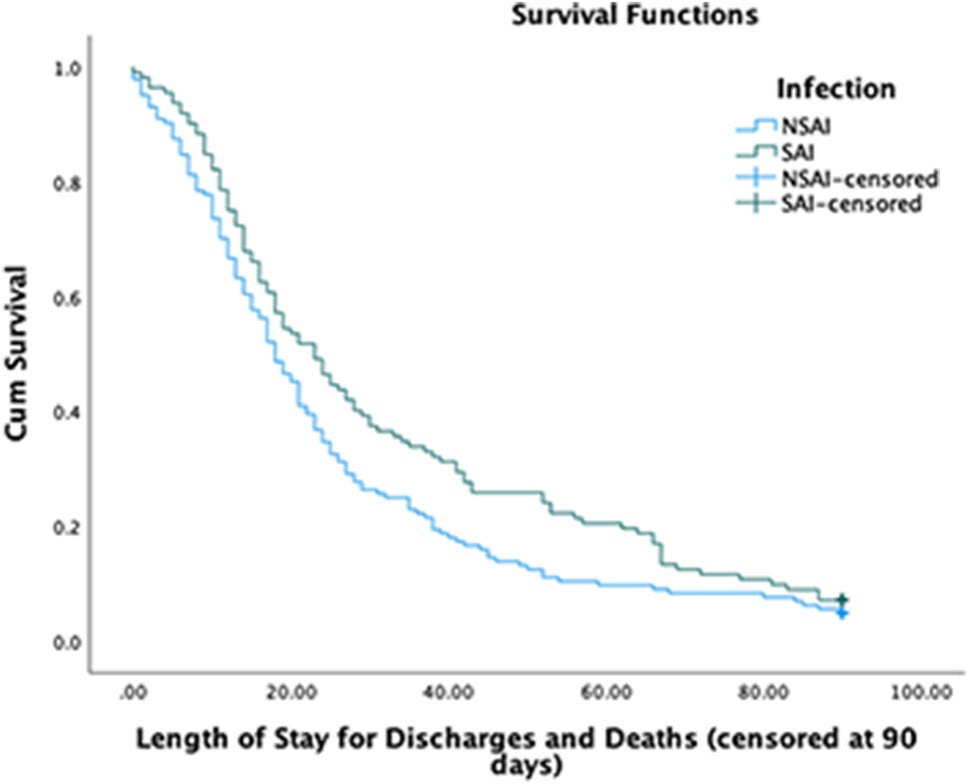
Kaplan Meier curve showing the length of stay from COVID-19 diagnosis until discharge or death, in the number of days, comparing the No SAI and SAI groups censored at 90 days. The Y axis indicates the proportion of patients yet to be discharged or die. *NSAI* No SAI group, *SAI* SAI group

**Fig. 2:**
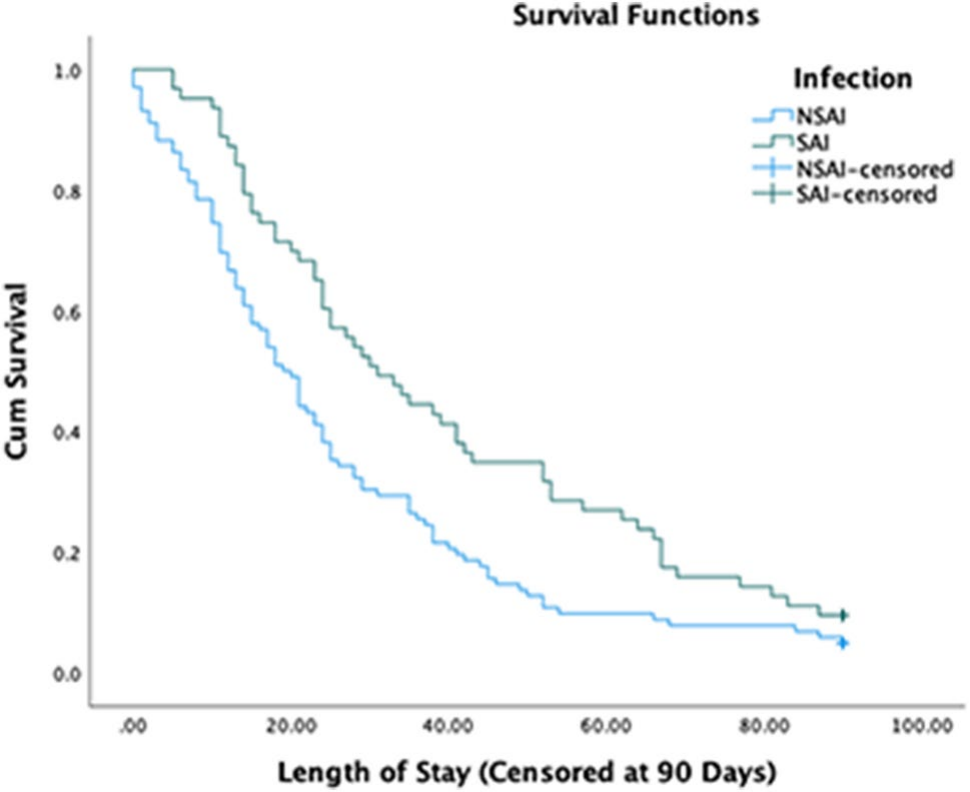
Kaplan Meier curve showing the length of stay from COVID-19 diagnosis until discharge, in number of days, comparing the No SAI and SAI groups censored at 90 days. The Y axis indicates the proportion of patients yet to be discharged. *NSAI* No SAI group, *SAI* SAI group

In the group with possible SAI, the 90-day mortality rate was higher than in the NSAI group (45.2 versus 30.7%, *p* = 0.020, Table 2). 30-day mortality was also significantly greater in the SAI group (55.7 versus 34.9%, *p* = 0.006, Supplementary Table 1). Patients with possible SAI had a longer inpatient stay (23 versus 18 days, *p* = 0.026, Fig. 1); longer still, when corrected for inpatient mortality (31 versus 19 days, *p* = 0.003, Fig. 2). When censoring at 30 days, length of stay remains prolonged in the SAI group (23 versus 18 days, *p* = 0.033, Supplementary Table 1) and is longer still when corrected for inpatient mortality (30 versus 19 days, *p* = 0.004, Supplementary Table 1).

**Table 2:** Patient outcomes and 90-day mortality

Outcome	All patients	Possible SAI	No SAI detected	*p*
90-day mortality rate	90 (36.9%)	47 (45.2%)	43 (30.7%)	0.020
Length of stay (days)	19 (27)	23 (50)	18 (22)	0.026
Length of stay (days, excluding inpatient mortality)	24 (31)	31 (48)	19 (26)	0.003

*IQR* Interquartile range

Data shown are *n* (%) or median (IQR). Length of stay was measured in days from COVID-19 diagnosis, with censoring at 90 days

Logistic regression analyses adjusted for age, sex, dexamethasone use, and possible SAI suggest that age and dexamethasone use are independently associated with the primary outcome of 90-day mortality, with both having statistically significant associations. Possible SAI and sex are not significantly associated (Table 3). Supplementary Cox regression analyses applying the above covariates also concluded that age and dexamethasone use are both significantly associated with a greater hazard of dying, whereas possible SAI and sex are not (Supplementary Table 2).

**Table 3:** Association between predictor variables and 90-day mortality

Predictor	OR	95% CI	*P* value
Age	1.059	1.019–1.100	0.003
Sex	1.025	0.578–1.819	0.932
SAI	1.701	0.964–2.999	0.067
Dexamethasone Use	3.989	2.196–7.246	< 0.001

*OR *odds ratio, *CI* confidence interval

Tabulation of regression data for predicting outcomes in older adult COVID-19 patients

**Fig. 3:**
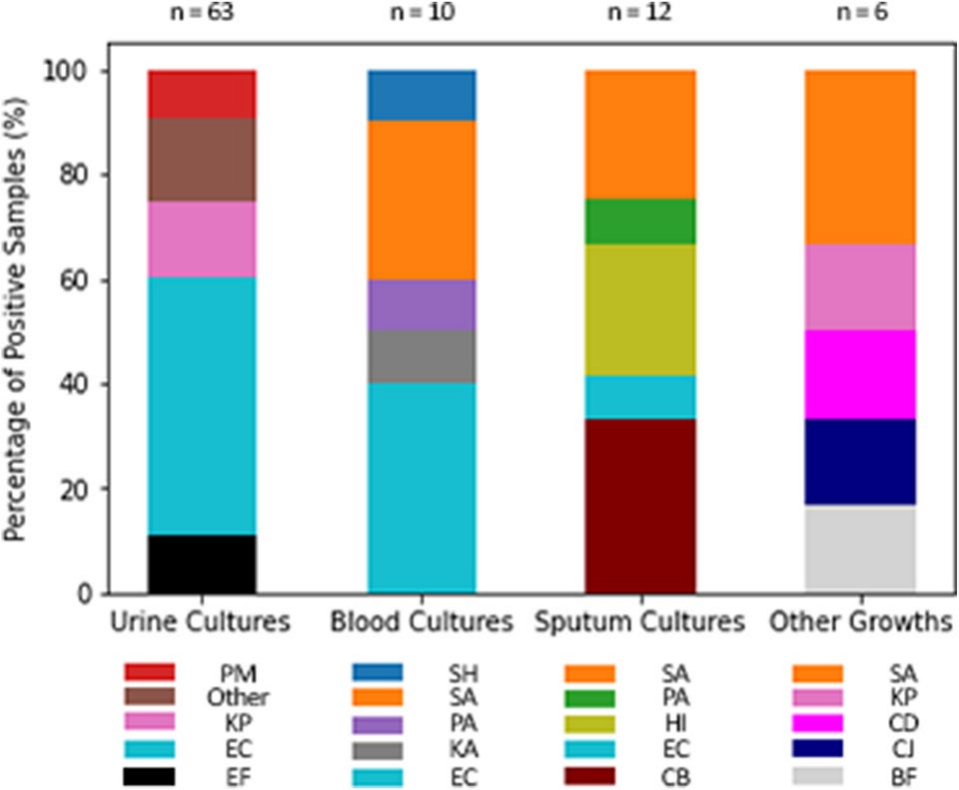
Bacterial growths observed in culture media. The Y axis indicates the percentage of growths within individual culture groups. Each culture type/bar is accompanied below by a reference guide of the organisms detected. *SH* Staphylococcus Haemolyticus, *SA* Staphylococcus Aureus, *PA* Pseudomonas Aeruginosa, *PM* Proteus Mirabilis, *PA* Propionbacterium Acnes, *KP* Klebsiella Pneumoniae, *KA* Klebsiella Aeruginosa, *HI* Haemophilus Influenzae, *EC* Escherichia Coli, *EF* Enterococcus Faecalis, *CB* Coliform Bacilli, *CD* Clostridium Difficile, *CJ* Campylobacter Jejuni, *BF* Bacteroides Fragilis

Of 63 bacterial growths recorded in urine cultures and 10 bacterial growths in blood culture, Escherichia coli (E. coli) was the most common pathogen (Fig. 3). Of 12 bacterial growths in sputum cultures Coliform bacteria were the most common. Other samples (skin swabs and stool cultures) recorded six bacterial growths.

## Discussion

We found that more than two in five older adults hospitalized with COVID-19 had investigations suggestive of SAI. Possible SAI was associated with increased mortality and length of stay. Microbiology suggested that E. coli was common in both urine and blood culture.

Our study was motivated by recent recommendations to limit the prescribing of antibiotics in patients presenting with systematic inflammatory response symptoms and COVID-19. The rationale behind the recommendation is sound, the virus could drive the presentation and indiscriminate use of antibiotics risks adverse effects and resistance. However, in an older adult population we were concerned that concomitant infection may be more prevalent, a concern supported by our data.

Multiple studies and systematic reviews have estimated bacterial co-infection to be infrequent in hospitalized COVID-19 patients [10, 11]. Although, when present, SAI was associated with a prolonged length of stay in hospital, an increased mortality rate, and a new ventilation requirement [11, 12]. The low prevalence of concomitant infection seen in other studies [3] contrasts with our data. This could partially be explained by age, with previous reviews including young to predominantly middle-aged populations, and selection bias with some studies not including those living with frailty [3]. Our population of older adults living with frailty, as measured by the 9-point clinical frailty scale [8] are more vulnerable to infection and adverse outcomes from any insult, including COVID-19 [9, 13].

Patterns of infection in our cohort were similar to younger groups, although absolute numbers with SAI were higher. For example, Karaba described a cohort with a median age of 62 years. Viral and respiratory tract infection, bacteremia, urinary tract infection (UTI) and other measures were investigated for 1016 patients over 5 acute care hospitals [14]. Evidence of UTI was reported in 30 patients (3%) whereas we reported evidence in 63 patients (24%), blood stream infections were reported in 20 patients (2%) compared to our 10 patients (4%). There were no substantial differences in patterns of microbiology when compared to a non-COVID older adult inpatient population [15]. In our cohort, comparing the time from admission to time of diagnosis of COVID-19 (Table 1) revealed that the SAI group had a greater median number of days in comparison to the NSAI group (17.5 versus 6 days, *p* = 0.006, Table 1). This could suggest that the SAI patient group in this study were being infected with a hospital acquired infection as they were inpatients for a longer period prior to their COVID-19 diagnosis.

Strengths of our analysis include the real-world nature of our data, and findings relating to potentially life-threatening disease in an under researched group. We acknowledge the weaknesses in our data. Primarily, we can only comment on possible SAI, as asymptomatic positive cultures, especially of urine, are likely. Within a medically unwell, older adult cohort there is no reliable method to confidently differentiate symptomatic and asymptomatic growths. Urinary symptoms such as dysuria and polyuria were not routinely recorded when data collection took place. It is also difficult to decipher whether lobar consolidation is due to a bacterial source or COVID-19. The most typical finding on plain radiographs of hospitalized COVID-19 patients is diffuse peripheral ground glass opacification, bilaterally [16]. This differs from bacterial pneumonia which more frequently presents with consolidation confined to a single lobe on CXR. However, it remains possible that a COVID-19 viral pneumonia could present as distinct single lobe consolidation. Our secondary results support presence of co-infection in the SAI group. For example, dexamethasone was prescribed for patients who were more unwell from a respiratory perspective, as would be seen in concomitant infection [17]. We restricted our data collection to a fixed period and may have lacked sufficient sample size to demonstrate modest between group differences, but our sample was sufficient to support our adjusted models.

Inpatient mortality and length of stay can be problematic outcomes, although they are often used in clinical COVID-19 research cohorts. Length of stay can be biased by early mortality and delays to discharge secondary to social care issues. In response some studies have looked at time spent at home, rather than length of stay [18]. Inpatient mortality assumes a direct relationship with the COVID-19 infection. Here there is a balance between assessing mortality too early and potentially missing COVID-19-related outcomes and assessing too late where the direct relationship with the index infection is less clear. We assessed mortality at 90 days as our primary outcome but also looked at 30-day outcomes. There was a statistically significant difference in mortality at the earlier assessment time, suggesting that COVID-19-related mortality is both immediate and protracted.

Despite UTIs being the most common source of bacterial infection in the > 65 adult population, UTIs carry a less significant mortality risk than bacteremia. Treatment for bacteriuria is often unprecedented in this patient population but symptomatic bacteriuria carries an increased risk of progression to urosepsis. Sepsis has a propensity for the older adult population and the likelihood of mortality in septic patients increases linearly with age [19, 20].

It is worth noting that procalcitonin was not routinely measured in the participating hospital. The favoured inflammatory marker in our study, CRP, showed no significant change. Procalcitonin can be used as a marker of severity of systemic inflammation due to a bacterial infection and may be more specific than CRP in diagnosing and monitoring sepsis [21]. Therefore, procalcitonin could have shown a significant difference between the SAI and NSAI groups. This would require confirmation in a further study. However, if proven, then procalcitonin may help in the initial assessment of the older adult presenting with COVID-19.

In conclusion, possible SAI is common in older adults hospitalized with COVID-19 and these patients experience worse outcomes. Our data do not change the advice to avoid antibiotics in most cases of COVID-19, but we feel we can make a strong argument for comprehensive assessment for other potential infection sources in this group.

## Supplementary Information

Below is the link to the electronic supplementary material.

Supplementary file1 (DOCX 14 KB)

## References

[1] Arentz M, Yim E, Klaff L, Lokhandwala S, Riedo FX, Chong M (2020). Characteristics and outcomes of 21 critically ill patients with COVID-19 in Washington State. JAMA.

[2] Ye Q, Wang B, Mao J (2020). The pathogenesis and treatment of the `Cytokine Storm' in COVID-19. J Infect.

[3] Langford BJ, So M, Raybardhan S, Leung V, Westwood D, MacFadden DR (2020). Bacterial co-infection and secondary infection in patients with COVID-19: a living rapid review and meta-analysis. Clin Microbiol Infect.

[4] Russell CD, Fairfield CJ, Drake TM, Turtle L, Seaton RA, Wootton DG (2021). Co-infections, secondary infections, and antimicrobial use in patients hospitalised with COVID-19 during the first pandemic wave from the ISARIC WHO CCP-UK study: a multicentre, prospective cohort study. Lancet Microbe.

[5] Quinn TJ, Mooijaart SP, Gallacher K, Burton JK (2019). Acute care assessment of older adults living with frailty. BMJ.

[6] Seaton RA, Gibbons CL, Cooper L, Malcolm W, McKinney R, Dundas S (2020). Survey of antibiotic and antifungal prescribing in patients with suspected and confirmed COVID-19 in Scottish hospitals. J Infect.

[7] Cuschieri S (2019). The STROBE guidelines. Saudi J Anaesth.

[8] Rockwood K, Song X, MacKnight C, Bergman H, Hogan DB, McDowell I (2005). A global clinical measure of fitness and frailty in elderly people. CMAJ.

[9] Hewitt J, Carter B, Vilches-Moraga A, Quinn TJ, Braude P, Verduri A (2020). The effect of frailty on survival in patients with COVID-19 (COPE): a multicentre, European, observational cohort study. Lancet Public Health.

[10] Lansbury L, Lim B, Baskaran V, Lim WS (2020). Co-infections in people with COVID-19: a systematic review and meta-analysis. J Infect.

[11] Garcia-Vidal C, Sanjuan G, Moreno-García E, Puerta-Alcalde P, Garcia-Pouton N, Chumbita M (2021). Incidence of co-infections and superinfections in hospitalized patients with COVID-19: a retrospective cohort study. Clin Microbiol Infect.

[12] Musuuza JS, Watson L, Parmasad V, Putman-Buehler N, Christensen L, Safdar N (2021). Prevalence and outcomes of co-infection and superinfection with SARS-CoV-2 and other pathogens: a systematic review and meta-analysis. PLoS ONE.

[13] Pulok MH, Theou O, van der Valk AM, Rockwood K (2020). The role of illness acuity on the association between frailty and mortality in emergency department patients referred to internal medicine. Age Ageing.

[14] Karaba SM, Jones G, Helsel T, Smith LL, Avery R, Dzintars K (2021). Prevalence of Co-infection at the time of hospital admission in COVID-19 patients, a multicenter study. Open Forum Infect Dis.

[15] Solís P, Vidales-Reyes M, Garza Gonzalez E, Guajardo-Alvarez G, Chavez-Moreno S, Camacho-Ortiz A (2015). Hospital-acquired infections in elderly versus younger patients in an acute care hospital. Int J Infect.

[16] Rodrigues JCL, Hare SS, Edey A, Devaraj A, Jacob J, Johnstone A (2020). An update on COVID-19 for the radiologist - A British society of Thoracic Imaging statement. Clin Radiol.

[17] The National Institute of Health and Care Excellence (NICE). COVID-19 rapid guideline: Managing COVID-19 2022. Available at https://www.nice.org.uk/guidance/ng19. Accessed 6 Jan 2022 34181371

[18] Quinn TJ, Dawson J, Lees JS, Chang TP, Walters MR, Lees KR (2008). Time spent at home poststroke: "home-time" a meaningful and robust outcome measure for stroke trials. Stroke.

[19] Gharbi M, Drysdale JH, Lishman H, Goudie R, Molokhia M, Johnson AP (2019). Antibiotic management of urinary tract infection in elderly patients in primary care and its association with bloodstream infections and all cause mortality: population based cohort study. BMJ.

[20] Martin GS, Mannino DM, Moss M (2006). The effect of age on the development and outcome of adult sepsis. Crit Care Med.

[21] Meisner M (2014). Update on procalcitonin measurements. Ann Lab Med.

