# Supplementary material for: Occurrence and outcomes of possible superadded infections in older adults with COVID-19—cohort study
Source: Eur Geriatr Med. 2022 Jul 13;13(5):1161–7. doi: 10.1007/s41999-022-00675-9 (PMC9553794; doi:10.1007/s41999-022-00675-9)
Supplement: Supplementary file 1 — Supplementary file1 (DOCX 14 KB) [file 41999_2022_675_MOESM1_ESM.docx]

**Supplementary Tables**

**Supplementary Table 1 – Patient outcomes and 30-day mortality.**

| **Outcome** | **All Patients** | **Possible SAI** | **No SAI Detected** | ***p*** |
| --- | --- | --- | --- | --- |
| 30-day mortality rate | 77 (43%) | 39 (55.7%) | 38 (34.9%) | 0.006 |
| Length of stay (days) | 19 (19) | 23 (18) | 18 (20) | 0.033 |
| Length of stay (days, excluding inpatient mortality) | 24 (18) | 30 (14) | 19 (20) | 0.004 |

*Data shown are n (%) or median (IQR). Length of stay was measured in days from COVID-19 diagnosis, with censoring at 30 days. IQR; Interquartile range.*

**Supplementary Table 2 – Result of Cox Regression**

| **Covariates** | **Coefficients** | **Standard Error** | **P-value** | **HR** | **95% CI for HR** |
| --- | --- | --- | --- | --- | --- |
| SAI | 0.070 | 0.214 | 0.744 | 1.073 | 0.705 - 1.633 |
| Age | 0.033 | 0.014 | 0.016 | 1.033 | 1.006 - 1.061 |
| Dexamethasone | 1.134 | 0.247 | <0.001 | 3.108 | 1.917 - 5.040 |
| Sex | 0.022 | 0.214 | 0.918 | 1.022 | 0.672 - 1.556 |

*Tabulation of multivariate Cox regression data for evaluating the effect of covariates on mortality in older adult COVID-19 in-patients. HR; hazard ratio, CI; confidence interval*
